# Translating Research into Clinical Scale Manufacturing of Mesenchymal Stromal Cells

**DOI:** 10.4061/2010/193519

**Published:** 2011-01-20

**Authors:** Karen Bieback, Sven Kinzebach, Marianna Karagianni

**Affiliations:** Institute of Transfusion Medicine and Immunology, Medical Faculty Mannheim, Heidelberg University, DRK-Blutspendedienst Baden-Wüerttemberg-Hessen, Ludolf-Krehl-Strasse 13-17, D-68167 Mannheim, Germany

## Abstract

It sounds simple to obtain sufficient numbers of cells derived from fetal or adult human tissues, isolate and/or expand the stem cells, and then transplant an appropriate number of these cells into the patient at the correct location. However, translating basic research into routine therapies is a complex multistep process which necessitates product regulation. The challenge relates to managing the expected therapeutic benefits with the potential risks and to balance the fast move to clinical trials with time-consuming cautious risk assessment. This paper will focus on the definition of mesenchymal stromal cells (MSCs), and challenges and achievements in the manufacturing process enabling their use in clinical studies. It will allude to different cellular sources, special capacities of MSCs, but also to current regulations, with a special focus on accessory material of human or animal origin, like media supplements. As cellular integrity and purity, formulation and lot release testing of the final product, validation of all procedures, and quality assurance are of utmost necessity, these topics will be addressed.

## 1. Mesenchymal Stromal Cells in Cellular Therapies

The vision for cellular therapies in regenerative medicine seems obvious: to replace diseased, dying, or missing cells or tissues with healthy cells [1]. The role of stem cells in this respect is under intense scrutiny, to define principles of organ regeneration and to develop innovative novel methods to treat organ failure. Mesenchymal stem or stromal cells (MSCs) emerge as key candidates for cellular therapies, covering regenerative and immune therapies. 

MSCs have a great appeal for cell and immune therapy and tissue engineering for numerous reasons:

1. they are relatively easy to procure from a variety of tissues [2];
2. they expand rapidly in cell culture [3];
3. they show only minor spontaneous differentiation during *ex vivo* expansion [4];
4. they are multipotent [4, 5];
5. they form supportive stroma for hematopoiesis and support hematopoietic stem cell engraftment [6];
6. they seem to be largely immunologically inert, paving the way for allogeneic transplantations [7];
7. they are immunosuppressive [8];
8. they secrete numerous trophic factors which modulate inflammation, remodelling, and apoptosis [9].

Based on the initial work of Friedenstein and Caplan, bone marrow-derived MSCs (BM-MSCs) are the ones best described and most advanced in clinical settings. In most comparative studies, BM-MSCs serve as gold standard [2]. For therapeutic applications, easily accessible and highly abundant sources are advantageous. Adipose tissue (AT), most often obtained as lipoaspirate, has emerged as an alternative tissue because cells occur at high frequency and the procurement is less invasive than that of BM aspiration [2]. Blood is obviously the most accessible adult tissue source for cells. Peripheral blood, however, does not contain MSCs in a nonpathological setting, at least not in numbers relevant for clinical scale manufacturing [10]. The same findings still hold true for umbilical cord blood (CB) [2, 11]. An inverse correlation between the gestational age and yields of MSCs is described [12]. Thus MSCs in full term CB are present at only low frequencies which hamper the isolation success [2, 11, 13]. In contrast to CB, isolation success is guaranteed when using the umbilical cord matrix or Wharton's jelly [14]. Fetal tissues are interesting as they seem to contain comparatively immature MSCs expressing pluripotency markers like SSEA- 3, and 4, Oct-4, Sox-2 and Nanog [15].

## 2. Towards Clinical-Scale Manufacturing of MSCs

MSCs are increasingly used in many preclinical as well as in some clinical settings for immunomodulation or tissue repair as summarised in more detail in the adjoining sections of this special issue. It is important to note that up to date administration of MSCs proved to be safe and also efficacious in a variety of disorders [16]. Several of these disorders are characterized by both inflammation and tissue defects. Often it cannot be dissected whether efficacy of MSCs is due to their production of trophic factors which stimulate endogenous repair mechanisms, their direct differentiation into various cell types or their immunomodulatory effects. In some of the models, factors released by the MSCs are obviously sufficient to mount a substantial part of the effect [9]. Thus whether efficacy of MSCs requires long-term persistence of MSCs remains to be elucidated.

Translating the rapid progress in stem cell science into innovative cellular therapies led to early and late stage clinical trials. However the complexity of the translational process ranging from the conception, to advanced clinical testing and finally new cell therapeutic drugs revealed a lag behind of standards and guidelines and often suffer from a regulatory burden. Increased scrutiny by the regulatory authorities is mutually dependent from the increasing numbers of cell therapy clinical trials. A variety of organisations responded to that threat to propose minimal set of standards or consensus guidelines [17–21].

Controversies about characteristics and potencies exist most probably due to the fact that different laboratories employ not only different tissue sources, but also extraction methods, culture protocols, and characterisation tools. Any variation may result in isolation and expansion of different subpopulations of cells or may change characteristics of the cells [22]. Thus Dominici et al. proposed minimal criteria defining MSCs [17], namely,

1. adherence to plastic under standard culture conditions,
2. expression of CD105, CD73, and CD90, and lack of expression of CD45, CD34, CD14, or CD11b, CD79alpha or CD19 and HLA-DR surface molecules,
3. differentiation to osteoblasts, adipocytes, and chondroblasts *in vitro*.

Given the fact that even MSCs used in clinical trials are produced and characterised by a variety of different protocols, reproduction or interpretation of the clinical results may be hampered [21]. Accordingly, standardized protocols have to be developed assuring that the manufactured cells behave solely in the clinically intended purpose and do not exert adverse effects by, for example, uncontrolled differentiation or transformation. Typically MSC can be cultured for 40–50 population doublings until the growth rate declines significantly and cells undergo replicative senescence [23]. Under certain conditions, however, MSCs have been observed to transform spontaneously. Discrepancies were resolved, when laboratories reported that transformation of MSCs was caused by cross-contamination with tumor cell lines [24, 25]. 

Therefore, an essential requirement is that all steps in MSC manufacturing from starting material up to potency testing for the intended indication have to be highly standardized to assure a required and reproducible cellular quality and potency. The challenge for scientists aiming at producing MSCs for clinical trials is to define optimal cell culture conditions to efficiently isolate and *ex vivo* expand homogenous MSCs while maintaining cellular qualities required for the intended clinical application and minimising risks of adverse events. For example, we recently verified whether AT-MSCs isolated and expanded in human serum (HS) share characteristics with cells cultivated in fetal bovine serum (FBS) [22, 26]. Although all criteria defined in [17] have been fulfilled, minimal differences were obvious regarding cell size and gene expression profiles. By comparing two human supplements to FBS, we observed that FBS alters gene expression slightly, but in genes categorized to differentiation and adhesion/extracellular matrix [22]. Current studies focus on dissecting in detail whether the supplements alter cellular behaviour in a way affecting the intended therapeutic application.

The therapeutic aim is to repair cell or tissue damage but without the risk of inducing tumors, severe immune reactions, or unwanted tissue development. Thus both safety and efficacy measures shall be considered in the establishment of the manufacturing process. Scarcity of MSCs often requires *ex vivo* expansion; extensive expansion in consequence may lead to ineffective or degenerated cells [23]. Thus it is important to understand and carefully control the production process and accordingly to define measures that reliably predict safety and efficacy of cell therapeutics.

## 3. Regulatory Frameworks

The employment of adult stem cell types in clinical studies, in general, necessitates formal approval by the respective regulatory body. This approval requires manufacturing, processing, and testing of cellular products according to the current national regulations, including current good tissue practice (GTP), good manufacturing practice (GMP) and good clinical practice (GCP). All cell-based products shall comply with these rules to ensure the product is safe, pure, and potent. GTP and GMP refer to common standards, regulating facilities, personnel, equipment, reagents and supplies, procedures and finally controls (process, final product and laboratory controls). These standards should be considered as soon as the development of a cellular product begins. For that reason, regulatory authorities offer investigators their advice to fine-tune the process from the early beginning on. 

In Europe, MSCs are classified as advanced therapy medicinal products (ATMPs) [27, 28]. ATMPs include gene therapy medicinal products, somatic cell therapy products (as defined in Directive 2001/83/EC), and tissue-engineered products. Cells fall under this regulation in case they have been subjected to substantial manipulation, resulting in a change of their biological characteristics, physiological functions or structural properties relevant for the intended therapeutic application, for example, regeneration, repair, or replacement. ATMP refers to cells or tissues that are not intended to be used for the same essential functions in the recipient as in the donor. This means that MSCs can be considered as somatic cell therapy products or tissue-engineered products depending on the indication and the manipulation during the manufacturing process. Concerning clinical trials with MSCs the rules set out in Article 6(7) and Article 9(4) and (6) of Directive 2001/20/EC shall apply. In the EU the responsible body for clinical trials approval are the health authorities at national level. This is in contrast to USA, where the NIH takes over this part [28, 29]. In the EU, GMP and GCP are more interrelated than in USA. The European Regulation No. 1394/2007 is effective since December 2008 and is binding in its entirety and directly applicable in all Member States. (Regulation (EC) No 1394/2007 of the European Parliament and of the council http://ec.europa.eu/health/human-use/advanced-therapies/index_en.htm. The main elements are (i) a centralised marketing authorisation procedure, (ii) the committee for advanced therapies (CAT) as multidisciplinary scientific committed to review the quality, safety and regulatory aspects of ATMP, (iii) technical requirements adapted to particular ATMP characteristics incentives for small- and medium-sized enterprises.) It is in compliance with the 2004/23/EC directive on donation, procurement and testing of human cells and tissues and with the directive 2002/98/EC on human blood and blood components. It is amending the Guideline on cell-based medicinal products (EMEA/CHMP/410869/2006) which focuses on the manufacturing and quality control of cell-based medicinal products as well as their nonclinical and clinical development. 

In the US, the FDA (Food and Drug Administration) announced in 1997 the “Proposed Approach to Regulation of Cellular and Tissue-Based Products” (21 CFR 1271). (http://www.accessdata.fda.gov/scripts/cdrh/cfdocs/cfcfr/CFRSearch.cfm?CFRPart=1271. Sec. 1271.1: “The purpose of this part,…, is to create a unified registration and listing system for establishments that manufacture human cells, tissues, and cellular and tissue-based products (HCT/P's) and to establish donor-eligibility, current good tissue practice, and other procedures to prevent the introduction, transmission, and spread of communicable diseases by HCT/P's”.) This became effective in 2005 as rules for Human Cells, Tissues, and Cellular and Tissue-Based Products (HCT/Ps). Although only one cell-based product (Carticel, autologous cell-based product for cartilage repair manufactured by Genzyme) has been licensed by the FDA, this does not reflect the actual number of trials with cell-based products. Those cell-based therapeutics do not require FDA-approval “that are minimally manipulated, labeled or advertised for homologous use only, and not combined with a drug or device” as specified by Parson, [30]. In contrast, manipulated autologous cells for structural use meet the definition of somatic cell therapy products and require an “investigational new drug” (IND) exemption or the FDA-license approval. In 2007 the “Guidance for Industry: Regulation of Human Cells, Tissues, and Cellular and Tissue-Based Products (HCT/Ps)—Small Entity Compliance Guide” and in 2009 the “Guidance for Industry on Current Good Tissue Practice (cGTP) and Additional Requirements for Manufacturers of Human Cells, Tissues and Cellular and Tissue-based Products” (http://www.fda.gov) have been released. The FDA provides recommendations to support manufacturing establishments of HCT/Ps to better understand and comply with the regulatory framework. Clinical studies employing MSCs underlie the IND mechanism. Accordingly investigator have to make an IND application, which necessitates detailed study protocols, describing the clinical plan as well as the preparation and testing of the therapeutic cell product [31]. 

Both regulatory frameworks in the EU and USA are to assure safety and thus they require a thorough analysis of all critical steps and aspects in advance. Although there are still differences [19, 28, 29], the authorities are in contact to further harmonise them. Thus it can be expected that by serving the requirements of one community, the chance is high to fulfil the others as well.

In the following parameters relevant for the manufacture of MSCs are exemplified.

## 4. Manufacturing Process

**Figure 1:**
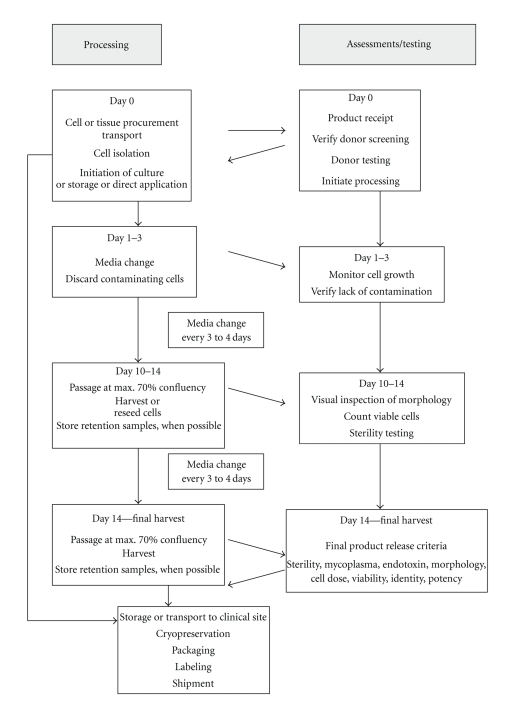
Flow chart illustrating essential processing and testing steps during MSC manufacture. This scheme summarises a GMP-compliant manufacturing process of MSC expansion separated into processing and related testing activities. On day zero the tissue is harvested and transported to the processing lab. Here donor eligibility criteria are checked again and donor testing/reception control initiated (viral, bacterial, blood group, condition, weight, if applicable cell counting, etc.). Verified reception control is a prerequisite for starting processing in the GMP facility. Here cell isolation is performed and expansion cultures initiated or the product is directly applied to the patient. Predefined in-process controls shall be taken at any critical processing step to verify cellular qualities and sterility. If expansion is initiated, normal protocols include a medium exchange step concomitantly depleting contaminating cells. Cell growth can be monitored visually as well as potential contamination. Passaging of the cells can be performed within the next 14 days involving controls for morphology, viability, and sterility. Assuming day 14 for the day of harvest and product release, cells have to be specified against predefined final product release criteria. The product can then be cryopreserved allowing for additional potency assays or directly transported to the recipient. Packaging, labeling, and shipment conditions again have to follow GMP rules.

The manufacturing process is highly fragmented as exemplified in Figure 1, illustrating a GMP-compliant MSC manufacturing process with processing and testing steps. Thus it should be well established and validated before initiating pivotal clinical trials because changes in the manufacturing process may confound clinical trial results. 

### 4.1. Tissue Procurement

In general, the starting material is a critical issue and includes common donor eligibility criteria, like age and viral testing. MSCs have been applied in autologous and allogeneic settings and derived from various tissue sources. Due to an immuno privileged status, a single allogeneic MSC donor may serve for multiple recipients raising the demand for well-defined eligibility criteria [32].

The most often used cellular source to obtain MSCs is BM, followed by AT and then other tissue sources, where we will herein focus on perinatal tissues. BM-derived MSCs are harvested via BM aspiration after puncture of the donors iliac crest. Aspiration strategy and volume impact the yield of MSCs, so that multiple aspirations from the same site and low aspiration volume (<8 mL) should be avoided [33]. Further hints indicate that donor age impacts the cell yield and the differentiation potential of MSCs [34, 35]. Aspirated BM volume can be a critical issue as high volumes can result in dilution with blood, too low volumes however demonstrated low to highly heterogeneous yields of MSCs [33]. Some data indicate that MSCs can be isolated from the marrow filter washouts dedicated for hematopoietic stem cell procurement [36]. For isolation, most protocols employ density gradient centrifugation, although the necessity for this step is debated and still under optimisation [37]. Mononuclear cells are then cultivated in MSC culture media until fibroblastoid cells show outgrowth.

AT represents an accessible source of MSCs, often referred to as adipose stem cells (ASCs) [38]. AT can be procured by different techniques, including excision or aspiration, from regions of the body where it is largely present (abdomen, trochanter region, groin, knee). MSCs can be isolated from the tissue by collagenase digestion and centrifugation and cultivation of the stromal vascular fraction to give rise to MSCs [2]. Various studies analysed the impact of harvesting conditions to the cell yield. The yield of ASC does not seem to be affected by the aspiration technique comparing syringe-based or pump-assisted liposuction [39], still there seem to be significant differences between the harvesting sites concerning the adipogenic properties [40] and their susceptibility to apoptosis [41]. Regarding the yield of the harvested ASC, there are controversial results: some report a richer yield of nucleated cells and colony forming units in hip versus abdominal liposuction [39], some others a superior yield of colony forming units after abdominal liposuction compared to hip liposuction [42].

In studies comparing liposuction versus tissue excision, liposuction method turns out to be superior as the cell yield in aspiration material remains stable even after 24 h storage in contrary to the decreasing cell yield of excisates. In contrast to these results a latter study reveals a higher cell yield and viability after excision [43]. 

Concerning the influence of the negative pressure during liposuction negative pressure of −350 mmHg leads to a greater cell yield than lower pressure of −700 mmHg [44].

Postnatal gestational tissues inherit numerous advantages over MSCs derived from aged adult tissues. Early focus on perinatal tissues harbouring stem cells arose from HSC and MSCs identified in CB [11, 45]. Subsequently fetal liver, lung, brain, but also villous placenta, fetal membranes as well as amniotic fluid were identified to host MSCs [13, 15]. Not discussing abortal tissues, in the majority of cases perinatal tissues are discarded at birth, thus cells harvestable without any risk for the baby or its mother. Consequently, there is an unlimited supply, easy access, and minimal ethical/legal issues associated with perinatal tissues. Tissues can be stored for autologous use or allogeneic settings as fetal cells have been demonstrated to be immuno-privileged. Hence CB storage is one strategy widely followed in numerous countries, not only for allogeneic, but also for potential autologous applications [46].

### 4.2. Manufacture at the Bedside: Volume Reduction and Direct Application

Some therapeutic applications, for example, in cardiac cell therapy, use solely minimally processed tissues, like volume reduced mononuclear cells which can be performed at the patients bedside [47]. This suggests an attractive and probably cost and time reducing option for autologous therapeutic settings and fall under different regulations (the ATMP so-called “hospital exemption”) (“Any advanced therapy medicinal product, as defined in Regulation (EC) No 1394/2007, which is prepared on a nonroutine basis according to specific quality standards, and used within the same Member State in a hospital under the exclusive professional responsibility of a medical practitioner, in order to comply with an individual medical prescription for a custom-made product for an individual patient.” (EU regulation 1394/2007)). Due to the high precursor frequency especially AT can be processed at the patients' bedside [48]. Still there are different aspects that have to be considered in order to minimize the risks of cellular therapy [49, 50]. But minimally processed tissues contain a large heterogeneous mixture of stem, progenitor cells, and mature cells, thus of suboptimal composition [51]. A few studies comparing volume reduced to expanded AT-derived cell preparations revealed different results [52, 53]. Consequently, to the current date all clinical applications specifying the use of MSCs have been using culture expanded cells.

### 4.3. Manufacture in a GMP-Facility

#### 4.3.1. Isolation and Expansion

In order to fulfil the regulatory standards the manufacture of a cell product requires the use of safe and pure components and materials. If possible, licensed or GMP-grade reagents should be used, in case of research-grade reagents additional in-house testing may be required to ensure safety and quality. Reagents, including supplements, cytokines, growth factors, used for expansion or differentiation of MSCs should be controlled and documented. Several parameters for *ex vivo* expansion of MSCs are critical to ensure both good expansion rates as well as maintenance of multipotency of MSCs. These include, for example, starting material, methods used for enrichment or separation, plating density, devices used for MSC culture, media, supplements and growth factors as well as passage number or population doublings [54].

#### 4.3.2. Cell Seeding

Plating densities have emerged as a critical issue for MSC isolation and expansion. Low seeding densities in primary culture seem to be associated with the emergence of more immature progenitor subsets [3]. Moreover, seeding at low densities allows higher expansion rates. For scale-up, accordingly, two different protocols are proposed. In one protocol cells are seeded at nearly clonal levels. This allows expanding the cells to high cumulative population doublings within one passage, but requires large culture area [55]. The other protocol seeds cells at higher concentrations. Due to reduced possible population doublings, this procedure necessitates a second passage, which, however, facilitates efficient depletion of contaminating cells [56].

#### 4.3.3. Media and Supplements

Culture conditions shall retain or even accelerate regenerative and trophic properties of MSCs. The variety in protocols is immense and a standard has not yet been defined. The classical media composition consists of a basal medium (DMEM or alpha-MEM) and 10%–20% supplement, most often FBS, which is available in GMP grade allowed for clinical use. The ongoing debate regarding xenogenic, especially ruminant proteins in pharmaceuticals also applies to MSCs. FBS bears the risk of transferring xenogenic, potentially infectious, or immunogenic proteins. Immunogenicity against FBS proteins has demonstrated to compromise the therapeutic benefit [57, 58]. Thus although GMP-compliant FBS batches are available and used in clinical grade manufacturing the regulatory authorities ask to replace FBS with a nonxenogeneic alternative if possible for the manufacturer. 

Up to now no completely serum-free media formulation in clinical-grade is available which allows both isolation (critical issue: attachment factors) and expansion of MSC [59]. Serum proteins provide not only nutrients but also essential attachment factors. Several laboratories have proposed the use of human components to supplement MSC growth medium. Here either autologous or allogeneic HS or platelet-derived factors have been evaluated [26, 54, 59, 60]. HS as well as platelet lysate are very crude protein cocktails. Essential growth factors for optimal MSC culture have not yet been defined. PDGF, EGF, TGF-*β*, and IGF have been subjected to investigation. Basic FGF has demonstrated most promising effects in expanding MSCs whilst maintaining stem cell properties and reducing replicative senescence [61]. Recently, Pytlík et al. described a HS and growth factor supplemented clinical-grade medium which allowed for high cell expansion mediated by loss of contact inhibition [62]. 

Any significant change in the production process may affect cellular functions. Accordingly, it is necessary to analyse the qualities of MSCs in comparability studies to ensure that cellular qualities are not compromised. Within a variety of publications, pooled human platelet lysate emerged as suitable alternative for BM-MSC isolation and expansion [26]. Our own data indicate that for ASC in contrast pooled HS has better effects on expansion [60]. But as specified above, it has to be determined whether therapeutic qualities are modified, improved or impaired. It is conceivable that depending on the clinical setting different protocols (cellular sources, manufacturing protocols, quality control/potency assays) come into place to derive the optimal product.

#### 4.3.4. Devices for Expansion

MSCs grow as adherent cells until reaching confluency and then further expand by serial passaging. Therefore the number of cells which can be harvested in an *ex vivo* expansion culture is determined by the surface area. Typically MSCs are cultivated in conventional monolayer cultures. In order to achieve a large surface area multilayered cell factories are used [55, 56]. This approach is labour intensive and money consuming. Also by using bioreactors it became possible to expand MSCs [63, 64]. As closed systems should be preferred in a GMP-setting, Rojewski et al. report a fully automated bioreactor allowing large-scale GMP-compliant manufacturing [65].

A critical issue is the proliferative age of MSCs: MSCs have a restricted lifespan and reach a senescent state in which cellular functions become diminished and the risk for accumulating mutations rises [32, 66]. Most often proliferative capacity is expressed by passage numbers. Passage numbers in contrast to population doublings do not describe the de facto proliferation history which is critical when reaching a certain—not yet well—defined time point (maximum 30 population doublings) [66].

#### 4.3.5. Storage or Cryopreservation

After isolation, volume-reduced cells or *ex vivo*-culture expanded cells can be transplanted directly or stored for long term under low temperature conditions. A variety of studies investigated the effects of storage conditions and cryopreservation methods and media [67–69] demonstrating that MSCs can be cryopreserved and thawed without loss of function [70]. Cryopreservation gives the only opportunity to perform time-consuming release tests prior to clinical application of the cells, hardly possible to achieve when the cell product is intended for immediate release.

### 4.4. Product Specification

As always it is necessary to weigh carefully the risk against the potential benefits of stem cell therapy. Potential risks can be reduced when applying appropriate release tests capable of ensuring safety, efficacy and consistency of the product. Cell-based products require special considerations on the manufacturing process, especially when they have to be applied immediately. In this situation it is impossible to obtain results of laboratory tests prior to the cell application, thus a limited set of controls has to ensure that the product fulfils all the predefined quality criteria. For cellular products in general sufficient numbers of viable, high quality cells are required. These can be easily documented by simple, rapid cell viability tests. 

Cell-based products cannot be sterilized to avoid transferring infectious diseases. By using human and/or xenogenic material, there is a potential for adventitious agent contamination, thus testing for bacteria, fungi, mycoplasma and viruses should be performed. Regardless of the use in autologous or allogeneic settings an increased attention to assure aseptic processing is mandatory. 

As indicated above, safety might be affected as prolonged *ex vivo* culture can accumulate aberrations. But only anecdotal studies indicate that MSCs may undergo spontaneous transformation, associated with chromosomal aberrations, induction of oncogenes and tumorigenicity after transplantation [23, 71]. Clinical experience indicates that cells, when harvested before onset of senescence, demonstrate extremely low probability of tumor formation [66]. Current testing systems including karyotype analyses, FISH, comparative genomic hybridization, or PCR to check for tumor marker expression may not be sensitive enough to detect the expected low proportion of affected cells [72], but even occurrence of alterations, like aneuploidity does not predict transformation, as recently demonstrated by Tarte et al. These data nevertheless helped to refine control assays, easily to perform, to control cell cycle/senescence and transformation pathways by, for example, PCR for p14, p16^Ink4a^, p21, p53, hTERT, and oncogenes like c-myc.

Further clinical safety concerns relate to possible ectopic tissue formation or other adverse events in the recipient. Despite the fact that in general no adverse reactions have been recorded, this possibility cannot be neglected yet. Mice treated locally with MSCs for myocardial infarction developed calcifications [73]. Further the relatively big cell size has been observed at times to cause pulmonary sequestration and embolism after intravascular transplantation [74]. Furthermore the protocol for cell application can affect transplantation as recently demonstrated when comparing different cell suspension media [75]. Finally, although therapeutically intended in some clinical settings, for example, to prevent or treat GvHD, the immunomodulatory capacities may also favour tumor growth or formation of metastasis, as observed in animal models [76–79]. 

For the EU and USA, the criteria for test procedures differ. Test procedures, for example, for sterility test, have to be approved by the FDA or the national regulatory authorities in Europe. But as the requirements are not identical, the international conference of harmonisation (ICH) intends to harmonise them (for more information, see respective homepages).

### 4.5. Identity and Impurities

The identity of cell-based products can be ensured where necessary by genotypic or phenotypic analysis. In MSC cultures, the fraction of cells displaying identity markers (mesenchymal markers) and the identification of contaminating cells (hematopoietic markers) can be easily and rapidly quantified by flow cytometric analyses [17, 80]. Above this morphological assessment of fibroblastic phenotype and proliferation can be easily documented in expansion cultures.

Product-related impurities have to be determined and specified, including endotoxin testing [81]. Where appropriate, impurities relating to, for example, degradation products from structural or matrix components shall be specified, as well as process-related impurities derived from added bioactive components.

### 4.6. Potency

Product characterisation has to consider the functional capacities related to the intended clinical use. The minimal criteria of the ISCT require to control the capacity of MSCs to adhere to normal plastic culture surfaces, to generate cells with a fibroblastic phenotype, which express or fail to express a typical set of surface markers, and to exhibit multilineage differentiation potential, at least into the osteo-, adipo-, and chondrogenic lineage [17]. Although every laboratory employs these assays, the assays are time consuming and far from being standardized yet, so comparison between laboratories and also the read-out of clinical data is hampered [80]. As mentioned before development of preclinical efficacy tests in the investigated indication are highly desirable as MSCs appear to employ different modes of function according to the intended use. Depending on the clinical intention, the following assays can be performed to assess potency, but it has to be kept in mind that none of these assays has been directly correlated to therapeutic efficiency [32].

**Clonogenicity**

The CFU-F assay is a suitable but not standardized tool to quantify precursor frequencies. Analysis demands for appropriate dilution to clonal levels as CFU-F frequencies do not follow a linear regression correlated to the input cell number [54, 55].

**Differentiation Potential**

The multilineage differentiation potential is a hallmark of MSCs, but discussed in detail elsewhere [4, 5, 82]. *In vitro* assays can be performed using self-made or commercially available induction media. However, it is increasingly discussed whether and to which extent *in vitro* data correlate to *in vivo* differentiation potential [82, 83].

**Immunomodulatory Capacities**

The perspective of modulating immune responses against allo- and possible also autoantigens has rendered MSCs an attractive population of cells for immune therapies. *In vitro*, assays have been established to quantify the expression of surface molecules, such as HLA class I and II and costimulatory molecules. In cocultures with peripheral blood mononuclear cells, MSCs do not elicit an alloreactive response. Furthermore, when added as third party in mixed lymphocyte reactions or mitogen-driven cultures, MSCs dose dependently inhibit immune cell responses. Very low concentrations of MSCs however can stimulate immune responses [84]. While this has not yet been observed *in vivo*, too low numbers of MSCs transplanted may accelerate the immune response rather than mitigate it in GvHD or autoimmune settings.

**Hematopoiesis/Stromal Support**

The beneficial effects of cotransplanting MSCs in haematological settings have already been demonstrated [85]. This effect can be assayed *in vitro* in coculture experiments using hematopoietic stem cells and MSCs and thus may be an adequate quality control system for this therapeutic indication [86].

**Trophic Support**

In a variety of settings MSCs showed promising therapeutic effects even though the transfused cells were—if at all—only barely detectable in the injured organs. Recent data further demonstrated that especially secreted factors actively modulate debilitating local inflammatory reactions. Reduction of apoptosis, and fibrotic tissue remodelling as well as recruitment of local resident regenerative cells contributed to the beneficial effects [9, 87]. Accordingly some studies already demonstrate therapeutic effects when infusing MSC conditioned medium instead of cells [88, 89]. Depending on the therapeutical setting, analysing the secretome by quantifying levels of chemo- or cytokines may subsequently emerge as additional potency assay [26, 60].

### 4.7. Validation

Safety and efficacy of a cellular product has to be demonstrated prior to their administration in humans. MSC application in clinical settings has progressed fast with 128 hits entering “mesenchymal stem cells” as search term under http://www.clinicaltrials.org. Nevertheless, there is a trend to go back from bedside to bench to better characterise and improve MSCs and importantly to standardise protocols for isolation, expansion, and finally characterisation. Changes *in vitro* necessitate translating them into clinical protocols. Thus it is a critical point to coordinate the clinicians needs with the researchers option in close interaction with the manufacturing laboratories, who overview the margin framed by the regulatory authorities. Any significant changes in the manufacturing protocols require validation *in vitro* and in appropriate* in vivo* animal models to assure safety and efficacy [28] as well as absence of toxicity (related to dedifferentiation or unwanted differentiation, migration to unwanted sites). All animal models have inherent limitations, like, for example, the application of human cells in a xenogenic milieu [82]. This requires the use of severely immunocompromised small animals preventing contrariwise analysis of immunological reactions. Furthermore for a variety of disease models, for example, in orthopaedics, small animals are not capable of modelling the disease. Consequently, one has to define a compromise between all requirements: clinical, regulatory, and laboratory to agree on appropriate validation strategies.

## 5. Conclusion

In the recent years numerous advancements led to the employment of MSCs in a variety of therapeutic indications raising expectations and hope. Although numerous clinical trials have been initiated worldwide (http://clinicaltrials.gov), standardized protocols for isolation, expansion, and characterisation, especially those on GMP-grade, seem to lag behind. The sum of clinical studies has supported safety and efficacy by demonstrating absence of major side effects associated with success reports. The lack of conformity between manufacturing protocols however is considered as potential threat to further development of the field. The heterogeneity of isolation, expansion, and characterisation protocols remains as obstacle. Thus to ensure the success of MSC-based therapies, we regard as a major critical issue to standardise and harmonise translational protocols in order to develop manufacturing processes along-side with developing therapies and not thereafter.
